# Supplementary material for: Metformin Attenuates Neutrophil Recruitment through the H3K18 Lactylation/Reactive Oxygen Species Pathway in Zebrafish
Source: Antioxidants (Basel). 2024 Jan 30;13(2):176. doi: 10.3390/antiox13020176 (PMC10886385; doi:10.3390/antiox13020176)
Supplement: Supplementary file 1 [file antioxidants-13-00176-s001.zip › antioxidants-2800762-supplementary.pdf]

Table S1. Primers designed for expression analysis in this study.

Gene	Gene ID	Note	Forward Primer (5′-3′)	Reverse Primer (5′-3′)
β-actin	NM_131031.2	qRT–PCR	ACGAACGACCAACCTAAACTCT	TTAGACAACTACCTCCCTTTGC
il-1β	NM_212844.2	qRT–PCR	GTACTCAAGGAGATCAGCGG	CTCGGTGTCTTTCCTGTCCA
il-6	NM_001261449.1	qRT–PCR	GCTATTCCTGTCTGCTACACTGG	TGAGGAGAGGAGTGCTGATCC
cxcl8a	XM_009306855.3	qRT–PCR	CCACACACACTCCACACACA	CCACTGAATTGTCCTTTCATCA
tnf-α	NM_212859.2	qRT–PCR	GCGCTTTTCTGAATCCTACG	TGCCCAGTCTGTCTCCTTCT
hdac3	NC_007125.7	qRT–PCR	CTATGGTGCTGGGCATCCAA	CCTCAGAGTGGAACCTGCAC
pkma	NC_007136.7	qRT–PCR	TGCAATGGCGGAAACCTTCT	AACGTGAAGCAGGTCCGAGT
gapdh	NC_007127.7	qRT–PCR	TCCTGAGCTCAATGGCAAGC	GGCAGGTTTCTCAAGACGGA
duox	XM_021470722.1	qRT–PCR	TCTTTCATCAGGAAAAACAGCAG	TCTCTCTGAGGCCTGACGTA
sod1	NM_131294.1	qRT–PCR	TCCACGTCCATGCTTTTGGT	CAGGTCTCCGACGTGTCTCA
cat	NM_130912.2	qRT–PCR	CCAGAAACGCATGGTGCAAA	GACGCTCCACCACGTGAATA
β-actin	NM_007393.5	qRT–PCR	CACTGTCGAGTCGCGTCC	TCATCCATGGCGAACTGGTG
Il-1β	NM_008361.4	qRT–PCR	CACTGTCGAGTCGCGTCC	TCATCCATGGCGAACTGGTG
Il-6	NM_001314054.1	qRT–PCR	TGCCACCTTTTGACAGTGATG	AAGGTCCACGGGAAAGACAC
Cxcr2	NM_009909.3	qRT–PCR	TCGTAGAACTACTGCAGGATTAAG T	ACAAGGCTCAGCAGAGTCAC
Tnf-*α*	NM_001278601.1	qRT–PCR	ACCCTCACACTCACAAACCA	ATAGCAAATCGGCTGACGGT
Hdac3	NM_010411.2	qRT–PCR	GCATTCGAGGACATGGGGAA	TTTCGGACAGTGTAGCCACC
Pkm	NM_001405491.1	qRT–PCR	TGTAAACCATCACCGGGTCTG	TTTGCCCTGAAGAGCTGGTTG
Gapdh	NM_001411840.1	qRT–PCR	GAAGGTCGGTGTGAACGGAT	ACTGTGCCGTTGAATTTGCC
Duox	NM_001099297.1	qRT–PCR	TGGCTTCTTTGGGAGCTCAG	GGTAGGGTTCTTTCAGGGGC
Sod1	NM_011434.2	qRT–PCR	AAGCGGTGAACCAGTTGTGT	GCACTGGTACAGCCTTGTGTA
Cat	NM_009804.2	qRT–PCR	AAGATTGCCTTCTCCGGGTG	GACATCAGGTCTCTGCGAGG
